# User experiences of evidence-based online resources for health professionals: User testing of *The Cochrane Library*

**DOI:** 10.1186/1472-6947-8-34

**Published:** 2008-07-28

**Authors:** Sarah E Rosenbaum, Claire Glenton, Jane Cracknell

**Affiliations:** 1 Norwegian Knowledge Centre for the Health Services, PO Box 7004, St. Olavs plass, N-0130 Oslo, Norway; 2 Rigshospitalet, Dep. 33-4-52, Blegdamsvej 9, 2100 Copenhagen, Denmark

## Abstract

**Background:**

Evidence-based decision making relies on easy access to trustworthy research results. *The Cochrane Library *is a key source of evidence about the effect of interventions and aims to "promote the accessibility of systematic reviews to anyone wanting to make a decision about health care". We explored how health professionals found, used and experienced The Library, looking at facets of user experience including findability, usability, usefulness, credibility, desirability and value.

**Methods:**

We carried out 32 one-hour usability tests on participants from Norway and the UK. Participants both browsed freely and attempted to perform individually tailored tasks while "thinking aloud". Sessions were recorded and viewed in real time by researchers. Transcriptions and videos were reviewed by one researcher and one designer. Findings reported here reflect issues receiving a high degree of saturation and that we judge to be critical to the user experience of evidence-based web sites, based on principles for usability heuristics, web guidelines and evidence-based practice.

**Results:**

Participants had much difficulty locating both the site and its contents. Non-native English speakers were at an extra disadvantage when retrieving relevant documents despite high levels of English-language skills. Many participants displayed feelings of ineptitude, alienation and frustration. Some made serious mistakes in correctly distinguishing between different information types, for instance reviews, review protocols, and individual studies. Although most expressed a high regard for the site's credibility, some later displayed a mistrust of the independence of the information. Others were overconfident, thinking everything on *The Cochrane Library *site shared the same level of quality approval.

**Conclusion:**

Paradoxically, *The Cochrane Library*, established to support easy access to research evidence, has its own problems of accessibility. Health professionals' experiences of this and other evidence-based online resources can be improved by applying existing principles for web usability, prioritizing the development of simple search functionality, emitting "researcher" jargon, consistent marking of site ownership, and clear signposting of different document types and different content quality.

## Background

The value of evidence-based medicine (EBM) – using updated, relevant and trustworthy evidence to inform medical decisions is widely acknowledged [1]. Recently the British Medical Journal nominated EBM as one of the 15 most important milestones in medicine since 1840 [2]. Easy access to high quality research has the potential to improve patient care, but there are obstacles that face health professionals attempting to use evidence in their practice. In an Australian survey, physicians identified insufficient time (74%), limited search skills (41%) and limited access to evidence (43%) as impediments to making better use of research data [3].

Systematic reviews directly address several of these barriers, as their summarized form reduces the amount of time and search skills needed to access and appraise many individual studies [4]. A systematic review is a summary of individual studies addressing a clearly formulated question that uses systematic and explicit methods to identify, select, and critically appraise the relevant research, and to collect and analyse data from the included studies. The Cochrane Collaboration is an international organisation of volunteers dedicated to producing systematic reviews of rigorous methodological quality. These reviews are published in one of the databases on *The Cochrane Library *[5], a web site that has the potential to further simplify the task of finding trustworthy evidence. Additionally the Library hosts other databases for systematic reviews, health technology assessments and randomized controlled trials, making it a central online collection of varying types of evidence from a variety of sources.

Part of the mission of The Cochrane Collaboration is "to promote the accessibility of systematic reviews to anyone wanting to make a decision about health care". The organization also aims to produce reviews that are easy to read and understand by someone with a basic sense of the topic [6]. But does the Library web site support the Collaboration's goals of clarity and ease of use, as well as the overreaching mission of making evidence accessible for decision making? We wanted to explore this question through observing the experiences of health professionals using *The Cochrane Library*. We were interested not only in site-specific problems but also in issues that might be relevant to other web sites publishing collections of evidence-based content.

### User experience

**Figure 1:**
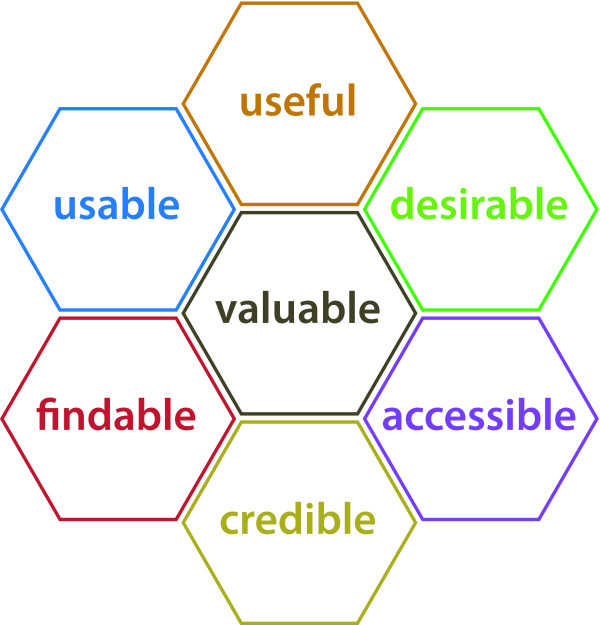
The honeycomb model of user experience, reproduced here with permission from Peter Morville, Sematic Studios LLC.

Usability testing is a method that is widely used in the field of web design to uncover errors and areas of improvement by observing users solving given tasks on the site [7,8]. There is increased recognition of the limitations of examining only task-related problems when attempting to understand why users' interactions with web sites might succeed or fail. Attention to the user's whole experience has begun to gain ground in the field of human-computer interaction [9]. Morville's "honeycomb" model (see Figure 1) distinguishes between seven separate facets of user experience, including findability, accessibility, usability, usefulness, credibility, desirability and value [10].

A brief explanation of these terms:

**Findability: **can users locate what they are looking for?

**Accessibility: **are there physical barriers to actually gaining access, also for people with handicaps?

**Usability: **how easy and satisfying is this product to use?

**Usefulness: **does this product have practical value for this user?

**Credibility: **is it trustworthy?

**Desirability: **is it something the user wants? Has a positive emotional response to?

**Value: **does this product advance the mission of the organization behind it?

Our study aimed to explore the user experience of health professionals trying to find evidence in *The Cochrane Library*, building on methods from usability testing. In this article we use the honeycomb model to organize findings from our study to illustrate more general potential pitfalls and challenges particular to evidence-based online resources. At the end we suggest some guidelines for designers, writers and developers working to improve the user experience of these types of sites.

## Methods

We carried out two series of user tests in 2005 (Test 1) and 2006 (Test 2), with participants from Norway and UK. The publisher of the site, Wiley-Blackwell, made changes to the site after Test 1, partly based on the results we uncovered. Most of these changes regarded branding at the top of the site, making *The Cochrane Library *the prominent identity and toning down the logo and universal navigation of the publisher. Therefore we altered the interview guide of Test 2 in small ways so that the questions would match the changes that had been made. See Additional file 1 for the complete interview guide we used in Test 2.

We limited our selection to health professionals who used the Internet and had some knowledge of systematic reviews, to ensure that the results of the interface testing would not be confounded by unfamiliarity with the media or the site's content. We sent email invitations to lists of previous attendees of evidence-based practice workshops, employees in the Directorate of Health and Social Affairs in Oslo and individuals in evidence-based health care networks in Oxford. Volunteers who responded were screened by phone or email to assess whether they fitted the requirements, and also to find relevant topics of interest so that we could individually tailor test questions. We also asked them about their online searching habits, and what sources of online information they usually used in connection with work. We did not reveal the name of the site we were testing during recruitment. Test persons were promised a gift certificate worth the equivalent of $80 USD or a USB memory stick if they showed up for the test.

Tests were performed individually and took approximately one hour. The test participant sat at a computer in a closed office together with the test leader who followed a semi-structured test guide. We recorded all movement on the computer desktop through use of Morae usability test software [11] and video-filmed the participant, who was prompted to think out loud during the whole session. We projected the filming of the desktop and the participant as well as the sound track, to another room where two observers transcribed, discussed, and took notes.

The data was anonymous to the degree that participants' names were not connected to video, audio or text results. We received written permission to store the recordings for five years before deleting it, guaranteeing that video/audio tapes would not be used for any purpose outside of the study and not be published/stored in places of public access. The protocol was approved by the Norwegian Social Science Data Services and found in line with national laws for privacy rights.

We began the test with preliminary questions about the participant's profession, use of Internet, and knowledge of *The Cochrane Library*. We then asked the participant to find specific material published on the Library starting from an empty browser window. Once on the site, we asked about their initial reactions to the front page, and they were invited to browse freely, looking for content of interest to themselves. Then we asked them to perform a series of tasks, some of which involved looking for specific content about topics tailored to their field or professional interests. For instance, a midwife was asked to find:

- all information on the whole library that dealt with prevention of spontaneous abortion

- a specific review about the effect of caesarean section for non-medical reasons

- all new Cochrane Reviews relevant to the topic "music used to relieve pain".

Other general tasks included finding help, finding the home page, and finding information about Cochrane. We also had specific tasks leading to searching and to reading a review. At the end, we asked if they had any general comments to the site and suggestions to how it could be improved.

Our analysis was done in two phases. The aim of the first analysis was to provide the stakeholders and site developers with an overview and a prioritizing of the problems we had identified. At least two of us carried out content analysis of the transcripts, independently coding each test. These codes were then compared, discussed and merged. The topics were then rated according to the severity of the problem for the user. We rated severity in three categories: high (show-stopper, leads to critical errors or hinders task completion), medium (creates much frustration or slows user down), or low (minor or cosmetic problems).

The second analysis was done to lift more generalizable issues underlying this article out of the site-specific data. We re-sorted the findings into the seven user-experience categories from the honeycomb model by re-reading the transcript, checking the context where the problems came from, and evaluating which of the seven categories best fit each finding. Severity-of-problem ratings from the first analysis were kept in the second analysis.

We did not evaluate accessibility (the degree to which the website complied with standards of universal accessibility, for instance as defined by the Web Accessibility Initiative [12]), since user testing methods are not an effective way of gathering data on various aspects of this issue.

The findings presented here are a selection of issues that received a high degree of saturation in our tests, and that we judge to be critical ("high severity") to the user experience of evidence-based web sites in general. This judgement is based on basic principles for web usability [7,13-15] as well as the principles underlying evidence-based health care: to successfully search for, critically appraise and apply evidence in medical practice [16].

Most of the findings here are still of relevance to *The Cochrane Library *in its current format, though we have included some observations of problems that are now resolved, because they illustrate issues that are potentially important for others. Our aim is not to write a critical review of the library, but to highlight issues we found that can be important to user experience of evidence-based web sites for health professionals.

## Results

### Participant profiles

We tested a total of 32 persons (See Table 1 for participant details). Test 1 included 13 persons from Norway, and Test 2 included five persons from Norway and 14 from the UK. Twenty-one of the 32 participants were non-native English speakers accustomed to reading in English.

**Table 1:** Participant details

	**Gender**	**Age**	**Profession**	**Internet use: ** **Frequency**	**Native ** **language**	**Place of ** **residence**
1	F	44	Midwife	Daily	Norwegian	Oslo
2	F	43	Sociologist, advisor in health-related govt. institution	Daily	Norwegian	Oslo
3	F	53	Physical therapist/teacher	1–2 times a month	Norwegian	Oslo
4	F	45	Midwife/researcher	Daily	Other (not English)	Oslo
5	F	-	advisor in health-related govt. institution	Up to 5 times a week	Norwegian	Oslo
6	F	-	Masters in nursing science, lectures at college level	Daily	Norwegian	Oslo
7	F	39	Midwife/teacher	Daily	Norwegian	Oslo
8	M	49	Medical Doctor/dept. director at health-related govt. institution	Daily	Norwegian	Oslo
9	F	28	Psychologist at health station for youth		Norwegian	Oslo
10	M	40–50	Medical Doctor/senior advisor at health-related govt. institution	Daily	Norwegian	Oslo
11	F	56	Sociologist/Masters in health admin./advisor at health-related govt. institution	Almost everyday	Norwegian	Oslo
12	M	25–35	Physical therapist	Daily	Norwegian	Oslo
13	F	28	Physical therapist at county health station	Up to 5 days a week	Norwegian	Oslo
14	M	43	Psychologist at hospital	Daily	Norwegian	Oslo
15	F	34	Medical Doctor at hospital	Up to 5 days a week	Norwegian	Oslo
16	M	49	Medical Doctor at hospital	Daily	Norwegian	Oslo
17	F	54	Midwife/teacher	3 times a week	Norwegian	Oslo
18	F	23	Nurse (recently graduated)	3 times a week	Norwegian	Oslo
19	F	42	Research nurse	5–10 hours a week	Danish	Oxford
20	F	-	Pediatric Nurse	10–20 hours a week	English	Oxford
21	F	45	Consultant, public health. Clinical dentist, doing an Mba	10–20 hours a week	English	Oxford
22	M	35	Medical Doctor	10–20 hours a week	English	Oxford
23	F	31	Psychiatrist	10–20 hours a week	English	Oxford
24	F	46	General practitioner	20–40 hours a week	English	Oxford
25	F	41	Mental Health nurse	5–10 hours a week	English	Oxford
26	M	66	Consultant Dentist Public Health	Less than 5 hours a week	English	Oxford
27	F	32	Nursing, Post-doc in nursing-related field	10–20 hours a week	English	Oxford
28	F	40	Clinical orthodontist	Up to 5 times a week	English	Oxford
29	F	45	Occupational therapist	Less than 5 times a week	Other (not English)	Oxford
30	F	50	Nursing, Midwife, starting Phd	Up to 5 times a week	English	Oxford
31	M	-	Dentist	Daily	English	Oxford
32	M	54	General practitioner	5–10 hours a week	English	Oxford

Participants were educated in nursing/midwifery (10); medicine (8); dentistry (4); physiotherapy (4); social sciences (3); psychology (2); and occupational therapy (1). They were currently working as health professionals in primary or secondary care (17); as government advisors working with health-related issues (7); as teachers at nursing/physiotherapy schools or universities (4); as research nurses (3); or as an editor for a patient information website (1).

Most used the Internet daily or several times a week, and much of this use was work-related. All had searched the Internet for health-related information or evidence. Most participants reported that they normally looked for information in response to a specific problem. A few of them had strategies to keep up to date within a certain field on a more regular basis. When in need of information, the most common sources mentioned were colleagues, research databases, and the Internet. All but one participant had some previous knowledge of The Cochrane Collaboration and 25 of the 32 participants could provide at least a basic description of the term "systematic review". Twenty-six said that they had visited *The Cochrane Library *site previously.

The findings that we included in this article are listed in Table 2.

**Table 2:** Main findings, sorted into the facets of the honeycomb user experience model


**Findability**	Difficulty finding the web site through Google or other external search
	Difficulty finding specific content on the site, using on-site search
	- non-English participants spelled search queries wrong
	- search engine too sensitive
	- keywords search didn't work properly
	- simple search produced unexpected results (i.e.: too few or too many of wrong type)
	- search results were misinterpreted, users confused document types
	- confusion when retrieving only a small number of search results
	Topics navigation not used or not seen
	Minimum of browsing even when encouraged to look around the site

**Usability**	Unfamiliar language/jargon caused confusion
	Text too small
	Too dense, too much text (front page, Help, More information pages)
	Important content too far down on page (review pages)
	Not interested in reading whole review
	Forrest plots unfamiliar and not intuitively located

**Credibility**	Users trusted content in The Cochrane Library
	Confusion about site ownership/neutrality due to dominance of publisher identity and universal navigation, weakens trust
	Misunderstanding about editorial quality evaluation – thinking all content on the whole site content has been reviewed by Cochrane

**Usefulness**	Assuming the library only dealt with medical topics (and not topics such as dentistry, nutrition, acupuncture)
	Misunderstanding targeted texts on front page, thinking content would be tailored for these groups
	Perceived as an academic resource
	Plain language summaries appreciated

**Desirability**	Site seemed off-putting, overwhelming
	Site can be alienating (research/academic identity and language)

**Value**	Felt Cochrane represented golden standard for systematic reviews
	Site is too difficult, would go elsewhere

**Accessibility**	Not evaluated

### Findability

#### Finding the website

Finding the site was an obstacle for the majority of participants in Test 1. Despite the fact that 11 of 13 of these participants said they had visited *The Cochrane Library *before, the same number were not able to find the site without considerable confusion, and six of these 11 did not find the site at all until they were helped by the test facilitator. Although most participants in Test 2 had more success, finding the site remained a problem for some. One of these, a EBM-skilled UK participant, used 23 minutes to arrive at *The Cochrane Library *from a blank browser page.

Much of this trouble stemmed from the participants' failed attempts to find Cochrane through Google search technology. These searches often failed because Google did not rank *The Cochrane Library *on the top of the first results page when queried for "Cochrane" or "Cochrane Library". In part this may be due to the fact that only the top few pages of *The Cochrane Library *were open to indexing in Google, affecting the ranking of the site. Several participants followed other links that appeared higher up on the results list, including links leading to the previous publisher of the site and to The Cochrane Collaboration site, expecting they would lead to the Library. After arriving at these other sites, participants continued to express confusion as to where they were because they found Cochrane-related content.

#### Problems searching for content

Finding specific content was also a major problem once participants arrived at *The Cochrane Library*. Participants attempted to solve most tasks by performing a search. Even when participants were asked to "take a look around the site", 75% started this task with a search. Few of our participants used the advanced search functionality. The simple search was the single most used feature in these tests, and many of these searches failed, leaving participants with a negative impression of the search functionality in the Library. Some participants compared *The Cochrane Library *to PubMed search, which they found easier to use.

Misspelling was the most common search-related mistake made by non-English participants. They were used to getting help with this from other search engines that was not provided by *The Cochrane Library *search: *"If I get the spelling wrong, Google will help"*. Another problem this group experienced was recalling precise terms (for instance recalling "overweight" but not "obesity"). The publisher redesigned parts of the search interface after Test 1. However in Test 2 the non-native English participants still had considerable problems finding content, mainly due to problems with spelling and recall of correct terms.

Search results were often misinterpreted. One of the most critical problems we observed was participants' confusion regarding what they were finding. Many participants did not notice that hits occurred in different databases in *The Cochrane Library *and thought all hits were completed Cochrane Reviews. We observed participants clicking on and reading review protocols and reports of individual clinical trials, mistaking them for systematic reviews.

The search engine was also too sensitive. For instance "huntingtons" gave no hits, while "huntington's" did. "Keywords" option did not provide stabile results.

Participants were also confused when their searches produced few or no search results. Some misinterpreted getting few hits as being the result of a bad search. The concept underlying the Cochrane Database of Systematic Reviews of one review per subject did not seem apparent. In addition, non-native English speakers interpreted a lack of hits as a result of their own bad English even though this might not have been the case.

#### Problems browsing for content

Test persons did not browse much, though this may have had to do with their problems understanding the organisation of the site. Few people were able to describe how the content was structured by viewing the front page and nobody could point to a menu with any certainty. Only one test person used the "Topics" entry at the top of the front page, though it was not apparent whether other participants did not see it or preferred not to use it.

### Usability

#### Language and jargon

Participants reacted to the use of jargon throughout the site. Some of the jargon was site-specific (such as the term "*record*" which led to full texts) and some was tied to research terminology (for instance "*protocol*"). The use of jargon gave the impression that the site was for academic use only and effectively discouraged participants from using several of the site's functions.

#### Legibility and layout

Most felt that there was too much text on the front page and that the type was too small. The participants that clicked on the "Help" and the "More Information" section also found them very dense.

"It's very messy. Do I have to read all of this?"

There was lots of frustration about the screen being taken up by other things than the review text such as the top banner space. Several participants made negative comments about having to scroll down to see full front page.

"The actual content is stuck in this little area down here."

#### Reading pattern

We were interested in how participants read reviews and asked them to show us how they normally would approach document if they had limited time (two to five minutes). Most referred to the conclusion section. Several said they would read the abstract, while some mentioned the objectives, results, and background sections. Most said that they normally would not be interested in reading a whole review.

We asked participants specifically about the forest plot graphs in the Cochrane Reviews, as they present a lot of information in a summarized form that could be useful for a reader in a hurry. Some participants found them helpful; others found them confusing. They were very difficult to comprehend for those participants who had not seen them before, and were not intuitively located.

### Credibility

When asked if they would trust the information on *The Cochrane Library*, all participants replied that they would, often because of a familiarity with the Cochrane name and more or less vague ideas about the quality of Cochrane products: "*because it's very respected*"; "*it's a reputable name*"; "*because I've heard good things about it*."

In Test 1, however, we observed potential challenges to this trust because of confusion about site/content ownership. This was primarily tied to the prominence of the Library's publisher Wiley-Blackwell on the website. Wiley's logo was placed higher up on the page than Cochrane's, and Wiley's Home, About Us, Contact Us, and Help buttons were assumed to be Cochrane Library buttons by most participants. Participants who used these buttons often did not realise that they were no longer in *The Cochrane Library*. When asked to describe the relationship between Wiley and *The Cochrane Library*, many described *The Cochrane Library *as a sub-group of Wiley:

"*It gives me sort of pharmaceutical industry associations. I think that The Cochrane Library is a subgroup (of Wiley)."*

Several changes were made to the website in order to address these issues after Test 1, and participants in Test 2 did not display the same confusion.

We also observed that *The Cochrane Library*'s perceived credibility could be over-interpreted. The only contents on *The Cochrane Library *that are "Cochrane approved" are the reviews listed in the Cochrane Database of Systematic Reviews. Despite this fact, some participants assumed that everything in the Library was "*Cochrane-approved"*, including the trials, reviews and reports in the individual databases: "*This will just have things that Cochrane have looked at*"; "*If I was looking for a piece of evidence and I found it on Cochrane I would think that it was high quality*."

### Usefulness

Some participants assumed that *The Cochrane Library *only dealt with medical topics and did not expect to find information on topics such as dentistry, nutrition, or acupuncture. The Library was also perceived by some as primarily an academic resource: *"I've tended to think that this is where researchers go to add to the body of knowledge or to see what there is, they'd use this (to build up) Clinical Evidence or Bandolier.... but if I was wanting to get back to the source of information, this is where I would want to go."*

The website has attempted to signal that it is a resource for all types of healthcare decision-makers by adding buttons on the front page entitled "For Clinicians"; "For Researchers"; "For Patients"; and "For Policy makers". These lead to short descriptions of what *The Cochrane Library *can offer each of these groups. However, while some participants thought these were advertising because of their position in the right-hand column, several others assumed that they led to specially adapted versions of *The Cochrane Library*, and were disappointed when this turned out not be the case:

"*I'm surprised that there's a link through to patients here. (...) I didn't realise that it was so well-developed along those lines."*

"Oh, so it's an (advert)... I was hoping it would give me a tailored search programme, a bit like NLH, which asks you "are you a GP..."

Others disliked these distinctions between different target groups: *"I don't know why clinicians should differ from researchers. We all need to have "high quality information at our fingertips."*

Several participants were positive to the fact that patients' information needs were being addressed in the form of the Plain Language Summaries they found in the Cochrane Reviews. They saw these products as helpful both for communicating with patients and for understanding the research results themselves.

"I wouldn't want to go and read all the nitty gritty. The short bits, the one page was useful."

### Desirability

Two thirds of the participants complained that the site looked messy and difficult to use, that there was too much information. All expressed frustration with failed attempts to find relevant content. Participants wanted a web site they could get into quickly, find what they were looking for, and get out again. "*Crowded*," "*busy*," "*cluttered*," "*a lot going on*," "*difficult to find any one particular thing*" were typical comments. Some participants felt "*overwhelmed*," "*bombarded*" and *"stupid."*

While most expressed interest in this type of evidence-based resource, many were cautious, or concerned that they lack the necessary skills: A nurse commented: "*This is maybe more for doctors*." A physician who had trouble finding specific content chose to search for *"dementia" *during a test task, and explained why: *"That's kind of how I'm feeling right now."*

### Value

At the beginning of the test all participants said they expected to be able to find content that was relevant for them on *The Cochrane Library*. Most felt that Cochrane Reviews represented the golden standard for systematic reviews. Many were put off by the amount of information and concerned about the time it would take them to find what they were looking for.

"Not easy to get around"; "Most of us don't have time to get around"; "So many pages are better designed, so you just get fed up and frustrated and go somewhere else."

## Discussion

Our study shows that health professionals' experiences of *The Cochrane Library *were considerably less than optimal. Test participants had much difficulty locating both the site and the evidence. Non-native English speakers were at an extra disadvantage when retrieving relevant documents. Many participants displayed feelings of ineptitude, alienation and frustration. Some made serious mistakes in correctly identifying different information types. Although nearly all expressed a high regard for the credibility of The Cochrane Collaboration, some later displayed a mistrust of the independence of the information. Others were overconfident, thinking everything on *The Cochrane Library *site had been quality-approved through an editorial evaluation, transferring the quality association they had of Cochrane Reviews to the entire content of the library.

There are few published usability studies of health professionals using online health libraries or other similar collections of evidence-based medical literature. A commercial company carried out parallel testing of *The Cochrane Library *for Wiley-Blackwell in 2005 and 2006. Their unpublished reports showed findings that were by and large similar to ours, though included only participants living and working in the UK and therefore did not duplicate the problems we found regarding non-native English speakers. One usability study of an NHS website published in 2003 [17] found that major problems were often caused by specialized library terminology. This supports our findings regarding unfamiliar language and jargon. The few other usability studies of health-related web sites we uncovered dealt with online information for patients or the public.

Our results were used in discussions with The Cochrane Collaboration Steering Group and the publisher, Wiley-Blackwell, in order to develop and improve The Library web site. Other publishers of evidence-based content could use the more generic results to improve their own websites.

### Searching (and finding): critical to evidence-based practice

*The Cochrane Library *site is not alone in having problems with findability. Results from usability tests of 217 web sites performed by Jakob Nielsen's team showed that search functionality and findability are the two largest categories of usability problems leading to task failure [7]. However, it is particularly ironic that a website built specifically to support evidence-based health care by synthesizing, organising and making accessible an overwhelming amount of health research should itself be perceived as overwhelming and difficult to navigate.

### Discriminating design

In this study the non-native English speakers, though displaying no visible trouble reading English text, were at an extra disadvantage when trying to search. Their problems were related primarily to difficulty recalling and spelling query terms that resulted in relevant hits. Creating a reliable base of evidence is a task no organisation or country can solve alone – cross-national efforts are needed. Easy access to a body of high quality evidence should not be limited to native English-speaking participants. There is a wealth of technology that could be used to improve the user experience of searching for non-native English speakers. Spelling aids or query translation from other languages would be particularly helpful to these kinds of users. Automatic query expansion with synonyms (used by PubMed) could provide a better experience both for all searchers but would be particularly helpful for those with a limited English vocabulary.

### Challenge – building a good mental model for evidence searching

Our findings revealed other challenges for designing good search functionality. In the Cochrane Database of Systematic Reviews, a precise query will result in only one or a few hits, as the underlying concept is one review per topic. However our participants' mental models of how search should function were based both on Google and PubMed, where simple queries produce a great number of results. The concept of a narrow search resulting only in a few hits is clearly still novel to many users and ways in which this can be made clearer need to be explored.

### Challenge – building a good mental model of evidence-based information hierarchy

Our findings showed that systematic reviews can be confused with protocols and reports of clinical trials, even among experienced users who have a clear idea of the difference between these document types. This kind of misinterpretation may happen especially when different document types are mixed together in search results lists. Different document types need to be distinguished from each other, both physically and visually – protocols should possibly be moved to a separate list. The importance of large clear labelling at the top of the individual documents enabling readers to easily distinguish between protocols, reviews and individual studies should also not be underestimated.

### Appraising the source instead of the document

A related problem is the tendency for users to assume all Cochrane Library contents are Cochrane-approved. Most of our test persons seemed inclined to be satisfied with a quality assessment short-cut: making judgements about the trustworthiness of the *publishing source *rather than critically assessing individual documents of research as EBM teaching encourages. This inclination, when coupled with poor signposting on a site containing information of varying levels of editorial evaluation and research quality, leaves a gap wide-open for serious misunderstandings about the strength and quality of different pieces of evidence. Blind trust of a whole source is a complex labelling and branding problem and needs to be addressed by publishers on many levels.

### Fragile credibility

Though Cochrane clearly enjoyed a high reputation among our participants, our study showed that even very small details can cause otherwise trusting users to suddenly question ownership and thereby credibility, such as an "About us" button leading to a page with a publishers' (unfamiliar) logo. While a large study from the Stanford Credibility project showed that consumers placed a lot of emphasis on the look of a site [18], a smaller parallel study showed that expert users tended to emphasize the reputation of the source when evaluating the trustworthiness of information found online [19]. Additionally it is important to follow the EBM principles of transparency and make it absolutely clear who is behind information that claims to be neutral and evidence-based.

### This site is not for someone like me...

Many of our participants felt that *The Cochrane Library *site was for *"researchers" *or others with more knowledge than themselves, in part due to use of unfamiliar or academic jargon, but also connected to their failure to find relevant information. The feelings of ineptitude expressed by participants in this study is perhaps mirrored in the Australian study, where 41% of the participating physicians blamed their own limited search skills as impediments to making better use of research data. In fact, many of the problems our participants encountered were not due to their own lack of skills, but to design flaws that could be solved following usability heuristics [20] and research-based guidelines for web design [7,13,21] or implementing better search technology. It is also important to signal inclusiveness and relevance to other health care areas than just medicine. Clear signs of content produced for patient target groups could also serve to lower the perceived threshold for professionals.

### Is valuable content enough?

Repeatedly we heard praise for the quality of content of this site. But frustration levels were very high, and several participants said they were ultimately too lazy to bother to use a site that made it so difficult for them. Information foraging theory describes user behaviour on the Internet as similar to wild animal's search for food: we want maximum benefit for a minimum of effort [22]. Jakob Nielsen points out that with the development of good search engines, it has become easier for information gatherers to move quickly between different hunting grounds, claiming that web sites should be designed less like big meals and more like tasty snacks, quick both to find and to eat [23]. A resource like Cochrane may be theoretically a great meal for a hungry animal, but too difficult to find and catch to be worth the effort, especially when less challenging prey is more easily available.

### Limitations of this study

Our goal is to identify the emerging issues rather than to quantify them. In reporting results, we have therefore not emphasized frequencies of events. As our data set has not been designed to statistically represent a set of respondents, presenting numbers can be misleading [24].

The user tests were performed in a laboratory setting, and may not reflect actual behaviour or reactions from real-life situations. For instance, increased time pressure in clinical situations may result in even higher degrees of user frustration when an interface does not easily or intuitively produce quick results.

UK-based tests were held in the office of The Cochrane Collaboration, and this may have influenced the answers of participants regarding use and attitudes towards *The Cochrane Library *and Cochrane Reviews, despite our assurances that we were not connected with the design of the web site. Answers regarding familiarity and use of research were self-reported and not empirically validated.

The honeycomb model was not used to design the interview questions, only applied in retrospect to our data analysis. This may have affected the relevance of the data we collected to this model. On the other hand, this may have led to less "leading" questioning on our part.

*The Cochrane Library*, like most websites, is under continuous development/change, and several of the weaknesses we identified have since been improved.

## Conclusion

### Recommendations based on findings

Building web sites for evidence-based practice is not much different than building for good web usability in general. However, the consequences of not finding information or of finding the wrong information have potentially critical consequences. Health professionals' user experience of evidence-based online resources can be improved by applying the following principles:

- Follow existing usability heuristics and web usability guidelines, designing especially for findability through search engines, as well as for speed of use particularly important to health professionals.

- If resources are limited, focus on improving simple (non-advanced) search functionality, including technology that will help non-native English speakers.

- Drop "researcher" language and jargon to encourage use by health professionals.

- Don't assume users possess good mental models of evidence hierarchies. Make document types evident where possible – through information architecture, labelling, and search results design.

- Clearly mark the difference between quality-approved content and not quality-approved content.

- Ownership and authoring must be clear at all levels of the site for supporting and maintaining credibility.

## Competing interests

CG is director of the Norwegian branch of the Nordic Cochrane Centre. JC is Co-ordinator & Managing Editor of The Cochrane Anaesthesia Review Group. CG and SR are involved in projects to improve summaries included in Cochrane Reviews. Test 2 was carried out in collaboration with Wiley-Blackwell, and partly funded by them.

## Authors' contributions

SR conceived of and designed the study, carried out user testing, data analysis and interpretation, and drafting of the manuscript. CG helped design the study, carried out user testing, data analysis and interpretation, and drafting of the manuscript. JC recruited UK participants, carried out user testing, transcribed and coded the British tests, and commented on the manuscript. All authors read and approved the final manuscript.

## Pre-publication history

The pre-publication history for this paper can be accessed here:



## Supplementary Material

Additional file 1 Appendix. Interview guide used in Test 2. Click here for file
