# Supplementary material for: User experiences of evidence-based online resources for health professionals: User testing of The Cochrane Library
Source: BMC Med Inform Decis Mak. 2008 Jul 28;8:34. doi: 10.1186/1472-6947-8-34 (PMC2529276; doi:10.1186/1472-6947-8-34)
Supplement: Additional file 1 — Appendix. Interview guide used in Test 2. [file 1472-6947-8-34-S1.doc]

**MANUSCRIPT – Test person nr. 1**

| **- Take test person to test room**  **- Go through the info page, any questions?**  **- Signature**  **- Hand out the ”payment” (USB-stick, etc.)** |
| --- |

**Test leader, prior to testing, check that these are done:**

| **- Erase ”cookies” and ”history” on the browser**  **- Take the browser window down**  **- Check that the previous test has been stopped and saved.** |
| --- |

**START**

| shorthand description | We’re going to take a look at a website that has been developed by the Cochrane Collaboration.  Our project has to do with seeing how user-friendly this web site is. Some of us have work which is connected to the Collaboration, but none of us have made this site, we’re just interested in seeing how usable it is. (So you aren’t stepping on our toes if you make critical comments.) |
| --- | --- |
|  | You are one of 15 people we’ve to help us evaluate this site. We will take the results from all of these tests to try to find out what parts need changing or more work, in order to make the site more user-friendly. |
|  | You and I will sit here in front of this screen. In another room, there are other people from Cochrane following the test. They see the same thing we do on the screen, as well as a small picture of the person behind the screen taken from the web cam. They can hear us as well.  They’re sitting in another room so that they don’t distract us. There will be a video tape made of this, as we explained earlier. But it will only be used in the ways we have described earlier. I hope this is ok? |
|  | This will take about 1 hour. I’ll be asking you some background questions about yourself and your use of the internett. Then we’ll spend most of the time looking at the actual web site. Afterwards I’ll ask you about how it was to use this site. You can take a break anytime you wish, and you can at any time decide to not participate any further. Ok? |

| **IMPORTANT! press “start” recording and check that the video icon is visible in the applications menu at the bottom of the screen (right hand side).** |
| --- |
|  |

**Background information**

| **A** | **Education/training background?** |  |
| --- | --- | --- |
|  | **Current position?** |  |

| **B** | **What year are you born?** |  |
| --- | --- | --- |

| **C** | **On an average, how often do you use the internet?** | |
| --- | --- | --- |
| Daily | |  |
| Up to 5 times a week | |  |
| Once a week | |  |
| Once-twice a month | |  |
| Never | |  |

| **D** | **In what context do you use the internet most?** | |
| --- | --- | --- |
| Private | |  |
| Work | |  |
| Studies | |  |
| Other | |  |

| **E** | **Apart from email, what do you use the internet mostly for?** | |
| --- | --- | --- |
| Prompting suggestions: | | |
| News | |  |
| Keeping up to date professionally | |  |
| Health info for personal/private use | |  |
| Ordering tickets | |  |
| Banking | |  |
| Games | |  |
| Getting information (phone numbers, yellow pages, etc.) | |  |
| Other… | |  |

**Internet experience relevant to this test**

Strategies for keeping up-to-date

| **F** | **In connection with your profession, do you try to keep up-to-date continuously or does it happen more sporadically when a problem comes up?** |
| --- | --- |
|  |  |

| **G** | **What do you do when you need to make decisions or give professional advice and you feel you don’t know enough to give a good answer?** |
| --- | --- |
|  |  |

Sources of information

| **H** | **Where do you usually go to get information when you have professional questions/uncertainties?** |
| --- | --- |
|  |  |

| **I** | **What do you think of the information you get there? Quality? Trust it?** |
| --- | --- |
|  |  |

Current information needs

| **J** | **Are you currently looking for/interested in any particular type of information these days in connection with anything at work?** |
| --- | --- |
|  |  |

| **K** | **(If yes): What kind of information are you looking for? And where would you go to look for it?** |
| --- | --- |
|  |  |

Forhold til Cochrane

| **L** | **Have you heard of Cochrane before?** |
| --- | --- |
|  |  |

| **M** | **Can you say something about what it is?** |
| --- | --- |
|  |  |

| **N** | **Do you know what a systematic review is?** |
| --- | --- |
|  |  |

| **O** | **Does the term evidence-based medicine mean anything to you?** |
| --- | --- |
|  |  |

| **P** | **Have you ever read a Cochrane Review or part of one before?** |
| --- | --- |
|  |  |

| **Q** | **Have you ever visited the The Cochrane Library web site before?** |
| --- | --- |
|  |  |

| **R** | **(If yes): When were you there last, and do you recall what you were looking for?** |
| --- | --- |
|  |  |

**Introduction to tasks:**

**Now I’m going to give you some tasks to solve.** What we’re looking at to see how easy or difficult it is to use the net site we are testing. By seeing how people really use the site, we can get an idea of what parts of the site need more working on to make it more user-friendly.

**We know from experience that those things you think are difficult, are difficult for many other people too.** We want your opinion and your experience, so there is no right or wrong answer to the things we ask. If you think something is easy or difficult, obvious or confusing, whether you can find things or cannot find things – we want to know about all of it.

Your job: to think out loud

**We’d like you to think out loud while doing the tasks.** Tell us both what you are doing and what you are thinking. For instance:

- Describe what you see on a new screen
- Tell us if it is what you expected to see
- Let us know when you are looking for something in particular, and when you find it
- Tell us why you are clicking on something
- If there are things you don’t understand – you can say for instance “I don’t know what this is…”
- Tell us when you are finished with a task or would normally not continue “Now I’m done” or “Now I would have given up.”

My role

**My role is to give you tasks and ask you questions. BUT –** since it is your opinion we are interested in, I will be saying as little as possible. You can ask questions, but I might not always answer them.

| **Open empty browser** |
| --- |

**Task 1: Finding a review from outside the library.**

| **1a** | - **Go to your normal “home” page,** the one that appears when you open your browser program at work. - **From here, we want you to find this Cochrane review about (see below), as if you had read about it in a newspaper and wanted to read more.** (Print out part of this press release (title and 4 paragraphs of text, don’t include the precise title of the review) and give it to test person:  **Contraceptive pills and patches don’t lead to major weight gain** (http://www3.interscience.wiley.com/homepages/106568753/PR_Contraceptives_and_weight.pdf) - **Just show us how you normally go about finding something like this.** if you were sitting in your office this morning and for some reason found that you needed this document. |
| --- | --- |

**Task 2: Assessing the site based on front page/first impression**

**If test person has found the Cochrane Library in Task 1:**

Go back to the front page of this site (then begin with question 2 below).

**If not:**

Find website

| **1b** | **Go to The Cochrane Library**  or  **Give them the url** [**www.thecochranelibrary.com**](http://www.thecochranelibrary.com/)  (depending on if we want to watch them finding the site or not.) |
| --- | --- |
|  |  |

First impression

| **2** | **Wait a bit before you click – what is your first impression of this site and the front page?** |
| --- | --- |
|  |  |

| **3** | **Do you think that you can trust the information you find here? What is it that leads you to believe that?** |
| --- | --- |
|  |  |

Understanding site objective

| **4** | **What sort of content do you think you can find here?** |
| --- | --- |
|  |  |

| **5** | **What do you think is the difference between this site and other sites you know of that are relevant to your profession?** |
| --- | --- |
|  |  |

Attention and expectations

| **6** | **What it is you notice here?  - anything that catches your interest or seems relevant to you?  - anything that surprises you? that you find confusing or didn’t expect to see here?** |
| --- | --- |
|  |  |

Overview, understanding the structure of the site

| **7** | **Can you describe the main areas of content of this site based on the front page?** |
| --- | --- |
|  |  |

Understanding the relation between Cochrane and Wiley

| **8** | **There are some logos on the top of the page (point to Cocrhane Library/Cochrane Collaboration logos and Wiley logo). Can you describe what you think the relationship between these are?** |
| --- | --- |
|  |  |

| **9** | **Which parts of the screen do you think have to do with Cochrane and which parts have to do with Wiley? (Look also at 2. level, under “Clinicians”)** |
| --- | --- |
|  |  |

Evaluation of web site based on front page

| **10** | **Do you think this web site has content that might be of interest to you?** |
| --- | --- |
|  |  |

**Task 3: Browse freely**

| **11** | **Take a look around this web site and see if you find anything relevant for you. Use the amount of time you normally would do visiting a site like this. Remember to tell us what you what you are looking at, what you are looking for, and what you’re thinking.**  (Test person may use a bit of time on this task, but should be interupted after 10 minutes, if they don’t stop by themselves. If they don’t seem to know how to begin, point them in a direction, such the Browse box. |
| --- | --- |
|  |  |

**Now I’m going to give you a number of tasks that entail finding specific things on the the site.** Remember that it is you who is testing the site, not the site that is testing you. There is absolutely nothing you can do wrong. If things seem confusing, or misplaced, or work in an unexpected or strange way – it isn’t your fault! Remember to talk out loud the whole time and tell me both what you are doing and thinking.

**Task 4: Find information about Cochrane**

| **12** | **Let’s say that a colleague asks you tomorrow about this test and wants to know more about The Cochrane Library. You can’t answer all his questions – can you find a page with general information you can point him to?** |
| --- | --- |
|  |  |

**Task 5: Browsing/searching by topic**

| **13** | **You want to find all the information there is on the whole web site on the topic: (specify topic relevant to test person here. For instance, for midwives we used “pregnancy and alcohol consumption”). How do you go about doing this? I want you to describe what you find – what sort of documents, how much you uncover, etc.**  **(to see if they understand the that there are other databases with more documenttypes here)** |
| --- | --- |
|  |  |

**Task 6: Looking for one specific Cochrane Review** (without having exact title)

| **14** | **You have read the following brief description of a specific Cochrane review in a professional journal:**  **(example for dentist test persons - taken from Plain language summary):**  Several therapies appear to either prevent or reduce the severity of mouth ulcers caused by chemotherapy or radiotherapy for cancer.  Treatment for cancer (including bone marrow transplant) can cause oral mucositis (severe ulcers in the mouth). This can cause discomfort, pain, difficulties in eating, and a longer stay in hospital. Different strategies are used to try and prevent this condition, and the review of trials found that some of these may be effective. Four interventions showed some promise. For patients with head and neck cancer these are amifostine, antibiotic pastille or paste and hydrolytic enzymes. For patients undergoing chemotherapy with 5-FU, ice chips may be effective.  **You want to find the whole review. How do you go about doing that?** (“Interventions for preventing oral mucositis for patients with cancer receiving treatment” – Oral Health) |
| --- | --- |
|  |  |

**Task 8: Look for all new reviews on a specific topic or field – or use protocol instead**

| **15** | **Consider if necessary – drop this if we need more time for next section**  **You want to keep update on a particular topic (find a relevant topic for this test person – for instance we used “prevention of spontaneous abort” for midwives) and therefore wish to look through all of the NEW Cochrane Reviews relevant to this. How do you find them?** |
| --- | --- |
|  |  |

| **16** | **Click on the “New” list.**  **Click on this title (point to a review that has a protocol icon).**  **First impression:**  **Describe your immediate reaction to what you see on the screen - what are you looking at, what your general feeling and impression is. What do you notice first? What would you actively be looking for first?** |
| --- | --- |
|  |  |

**Task 7: Search-related questions**

| **17** | **The starting point for this task is one of the searches you already have performed.** (Most likely, they have used search by now. Everyone in last test did by this point. Go to this point when they first use search and go through questions 17-22)**.**  **How many documents in total did this search recover from the whole site?**  (We want them to show that they have seen or not seen CRDB, DARE, CENTRAL, etc. |
| --- | --- |
|  |  |

| **18** | **Can you explain what these different categories are?**  (Point to the CRDB, DARE, CENTRAL parts). |
| --- | --- |
|  |  |

| **19** | **Can you re-organize the search result page in chronological order (so that the newest articles come first?)** |
| --- | --- |
|  |  |

| **20** | **Can you explain what you think the small icons next to the titles might mean?** |
| --- | --- |
|  |  |

| **21** | **Find “help” for searching** |
| --- | --- |
|  |  |

| **22** | **How do you get back to the front page from here?** |
| --- | --- |
|  |  |

**Task 9: Looking closer at one review**

| **23** | **Go to a review (for instance, from topics menu or from search results page) that is relevant to your field, but which you have not looked at before.**  **First impression:**  **Describe your immediate reaction to what you see on the screen - what you see, what your general feeling and impression is. What do you notice first? What would you actively be looking for first?** |
| --- | --- |
|  |  |

| **24** | **Can you tell us what this review is about?** |
| --- | --- |
|  |  |

| **25** | **What information is important to you if you have only 2-5 minutes?**  **Where would you expect to find that information? Show me where you would look.** |
| --- | --- |
|  |  |

| **26** | **Results of the review – show us all the places in the review where you think you could find results, presented in various formats or ways.** |
| --- | --- |
|  |  |

| **27** | **Look at this graf (click on “figure” at the bottom of the menu)  – how would you interpret this?)** |
| --- | --- |
|  |  |

Critical appraisal

| **28** | **What do you think about the quality of the review? What kinds of things do you base this opinion on?** |
| --- | --- |
|  |  |

| **29** | **How can you tell if the results of a review are relevant for a specific patient of yours. Which specific types of information would you be looking for? Show me where you find that information in the review or where you would expect to find it?** |
| --- | --- |
|  |  |

Integration into practice

| **30** | **If you found this review relevant, how would you go about using it or the content of it in your practice? Specific, detailed account of what you might do? (Print out, what parts, for whom and for what purpose)**  **- Which parts ?**  **- Would this evidence be enough, would they need other information?**  **- How would you communicate this evidence to a colleague?**  **- How would you communicate this evidence to a patient?** |
| --- | --- |
|  |  |

**DEBRIEFING**

**That was all of the tasks for today. Now we just have some questions to wrap it up.**

| **31** | **What kind of web site do you think this is? Why do you think it has been made?** |
| --- | --- |
|  |  |

| **32** | **How would you describe this site to a colleague with about the same level of internet experience as yourself?** |
| --- | --- |
|  |  |

| **33** | **Is this site an interesting source of information? Something you might use?** |
| --- | --- |
|  |  |

| **34** | **Did you find anything that was of real interest to you?** |
| --- | --- |
|  |  |

| **35** | **Do you think you found all of the information that might be of interest to you here?** |
| --- | --- |
|  |  |

| **36** | **Would you recommend this site to others? Why / Why not?** |
| --- | --- |
|  |  |

| **37** | **To summarize, can you say 3 positive things about this site, and 3 things you found to be negative?** |
| --- | --- |
|  |  |

| **38** | **OK, now that we have looked at the existing site, let’s brainstorm a little. Don’t think in practical terms at all, just do some wishful thinking. What might you wish a site or web service might do for you? Have you ever thought –“ I wish someone would invent a site or a service that would do such-and-such for me?”** |
| --- | --- |
|  |  |

**Do you have any final comments or questions?**

**That was all of the questions about the site.**

**Just one final question – do you have any suggestions as to how we might have done this test in a better way, for instance booking of your appointment, or the information you received, or the way the test was performed today?**

**Thanks very much. That’s all, we are finished.**

| **- Press “Stop” button to end the taping session.**  **- Give business card – they can email or ring later if they have any questions.**  **- Follow test person out.** |
| --- |

**After testing:**

| **- Erase ”cookies” and ”history” on the browser**  **- Take the browser window down**  **- Check that the previous test has been stopped and saved.** |
| --- |
